# Positive/retained SDHB immunostaining in renal cell carcinomas associated to germline SDHB-deficiency: case report

**DOI:** 10.1186/s13000-019-0812-6

**Published:** 2019-05-15

**Authors:** Marina Ugarte-Camara, Raul Fernandez-Prado, Isabel Lorda, Gabriela Rossello, Carmen Gonzalez-Enguita, Pablo Cannata-Ortiz, Alberto Ortiz

**Affiliations:** 1 Nephrology, Hospital Universitario Virgen del Rocio, Sevilla, Spain; 2 Nephrology and Hypertension, IIS-Fundacion Jimenez Diaz UAM and REDINREN, Madrid, Spain; 3 Genetics, IIS-Fundacion Jimenez Diaz UAM, Madrid, Spain; 4 Urology, IIS-Fundacion Jimenez Diaz UAM and REDINREN, Madrid, Spain; 5 Pathology, IIS-Fundacion Jimenez Diaz UAM and REDINREN, Madrid, Spain

**Keywords:** SDHB, Clear cell carcinoma, Hereditary renal cancer, Succinate dehydrogenase, PGL4 syndrome

## Abstract

**Background:**

According to WHO, succinate dehydrogenase (SDH)–deficient renal cell carcinoma is characterized by negative immunostaining for SDHB, which remains positive in non-tumor tissue despite germline mutations in the *SDHB* gene. We now report a patient with a *SDHB* mutation, *c.166_170del* (p.Pro56Tyrfs*5) who developed renal cell carcinomas with characteristic morphological features of SDH-deficient renal cell carcinoma but had positive SDHB immunostaining.

**Case presentation:**

Within a 6-year period, the patient developed two different renal cell carcinomas, which had characteristic morphological features of SDH-deficient renal cell carcinoma (uniform cells characteristically displaying eosinophilic granular material intermixed with fewer cells exhibiting clear intracytoplasmic inclusions and bland centered nuclei) but displayed immunohistochemistry for SDHB with a cytoplasmic granular positivity (mitochondrial pattern) in tumor cells. For the second case, this was initially interpreted as positive by IHC, but on review some subtle differences were identified.

**Conclusions:**

SDHB immunostaining may be positive in renal cell carcinoma associated to germline *SDHB* deficiency which have other typical morphological features. Immunohistochemistry interpretation may be complex.

**Electronic supplementary material:**

The online version of this article (10.1186/s13000-019-0812-6) contains supplementary material, which is available to authorized users.

## Background

The 2016 WHO classification of tumors of the urinary system and male genital organs recognizes succinate dehydrogenase–deficient renal cell carcinoma (SDH-deficient RCC) as a distinct entity [1, 2]. We now report a case of two SDHB-positive RCC associated to a germline *SDHB* mutation. Interestingly, SDHB immunohistochemistry showed a diffuse granular cytoplasmic positivity (mitochondrial pattern) in tumor cells, thus not fulfilling the 2016 WHO diagnostic criteria for SDH-deficient RCC, despite the characteristic morphological features and the association with a germline pathogenic *SDHB* mutation.

### Case presentation

Written permission to publish the case report was requested from the patient, who consented. Immunohistochemistry for SDHB was performed in an Omnis platform (Agilent Technologies) using a commercially available mouse monoclonal antibody (ABCAM ab14714, clone 21A11, dilution 1: 100), which is the same used in prior publications that allowed to define this pathological entity. A high pH antigen retrieval at 97 °C for 30 min was employed [3].

### RCC 1

A 29-year-old male, without any previous medical history, underwent a radical right nephrectomy in 2006 for clear cell RCC (eosinophilic variant) 8.5 × 7.5 × 5.5 cm. There was a family history of cancer, including larynx carcinoma and carotid body paraganglioma in his mother, and clear cell RCC in his maternal aunt.

### RCC 2

At age 35, the same patient underwent partial left nephrectomy for a renal mass, diameter 2.7 cm. The neoplasm was initially diagnosed as a hybrid oncocytic tumor but displayed similar morphological features than the previous contralateral RCC. Birt-Hogg-Dubé syndrome, also referred to as Hornstein-Knickenberg syndrome was suspected, but no *FCLN* mutation was found. However, the genetic analysis (see Additional file 1) disclosed a pathogenic deletion in exon 2 of the *SDHB* gene, c.166_170del (p.Pro56Tyrfs*5), which had been previously described [4, 5].

**Fig. 1:**
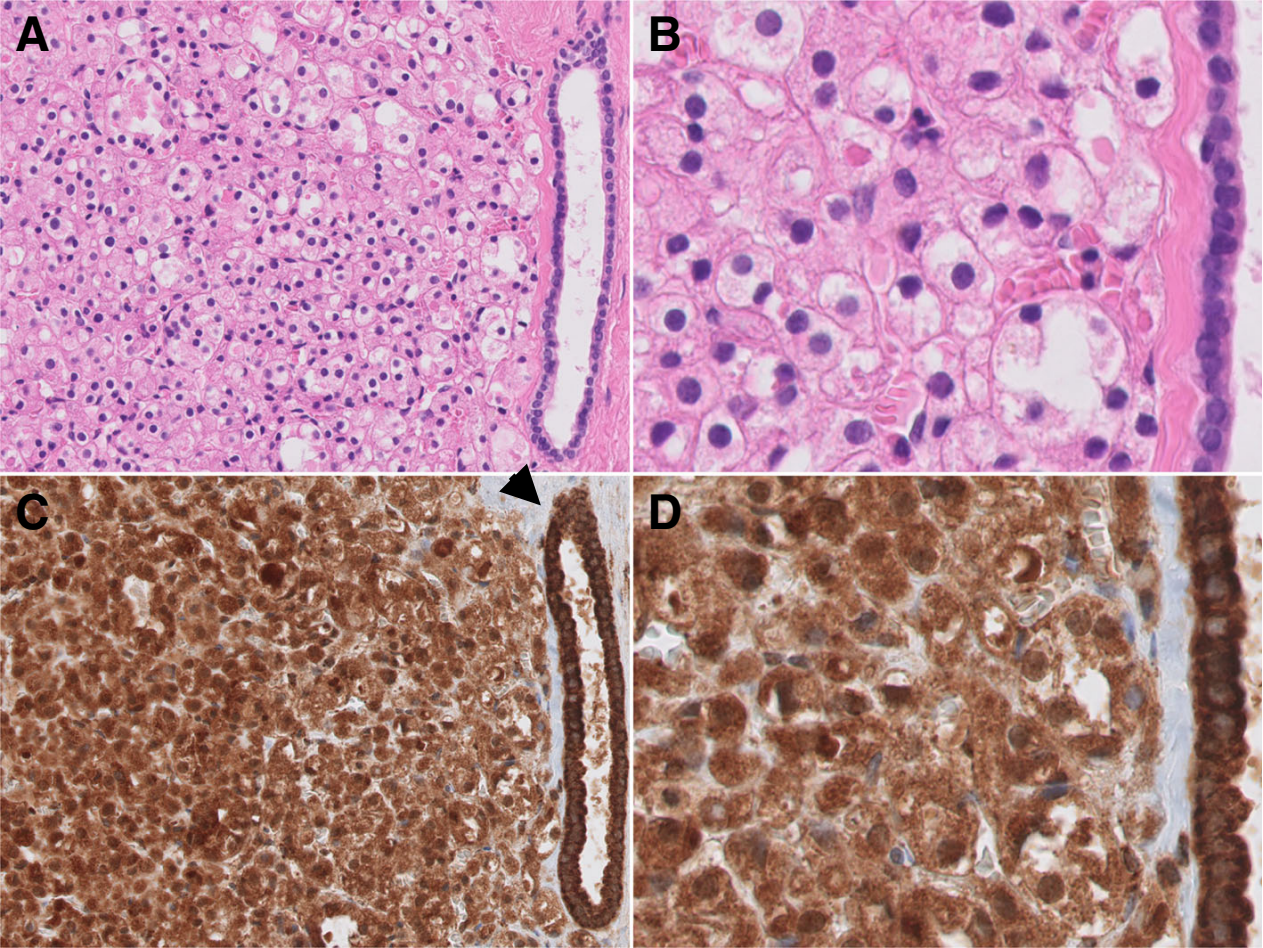
First renal cancer. **a** Tumor composed of solid sheets of uniform cells with granular eosinophilic cytoplasm intermixed with cells displaying a clearer cytoplasm. Entrapped tubules are identified at the periphery of the neoplasm (HE × 200). **b** Tumor cells display round and uniform nuclei. (HE × 400). **c** Tumor cells are positive for SDHB (SDHB × 200). **d** Note the granular positivity for SDHB immunohistochemistry in a cytoplasmic mitochondrial pattern. Non-tumor entrapped tubules show a stronger positivity for SDHB than tumor cells, arrowhead (SDHB × 400)

**Fig. 2:**
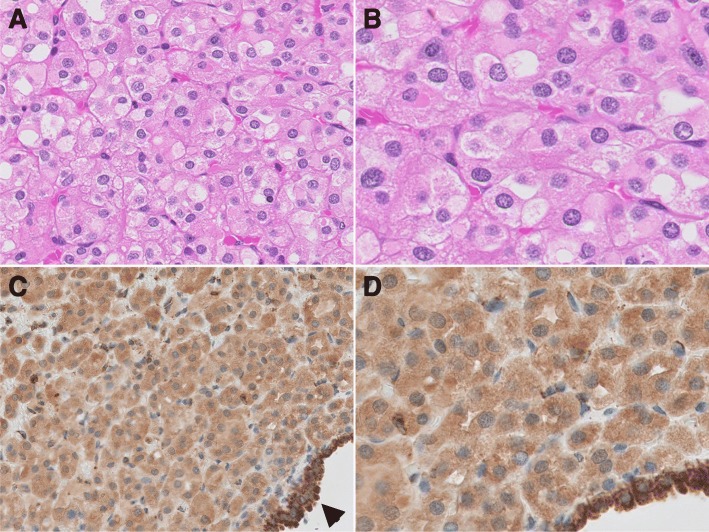
Second renal cancer. **a** Similar morphology to the previous tumor, composed oncocytic cells with different grades of eosinophilic cytoplasm (HE × 200). **b** Nuclei with evenly distributed chromatin (HE × 400). **c** Tumor cells stain positive for SDHB immunohistochemistry (SDHB × 200). **d** SDHB staining showing a cytoplasmic mitochondrial pattern. Just as in the previous tumor, entrapped tubules at the periphery are more intense for SDHB immunohistochemistry than tumor cells, arrowhead (SDHB × 400)

The histological features of both neoplasms were similar between themselves and to those previously reported for SDH-deficient RCC and were characterized by uniform tumor cells arranged in nests or solid sheets with a characteristic granular flocculent cytoplasm and cells with cytoplasmic pale inclusions which were vacuolated or contained eosinophilic fluid-like material [3] (Figs. 1a-b and 2.a-b). Occasional intratumoral cystic changes were observed. Non-neoplastic tubules were entrapped at the edges of these well-defined neoplasms with round borders. Tumors were EMA positive and negative for CK7, CK20, Vimentin and CD10.

Immunohistochemistry for SDHB disclosed diffuse intense granular staining in cytoplasm of non-neoplastic cells and a less intense but definite diffuse granular cytoplasmic positivity (mitochondrial pattern) in tumor cells (Figs. 1c-d and 2c-d). No areas of negative staining in tumor cells were identified throughout the different sections on SDHB immunohistochemistry. Tumor 2 was initially interpreted as positive by IHC, but on review for this manuscript some subtle differences were identified such as a blush cytoplasmic granular positivity in contrast to the more intense mitochondrial positivity seen on non-neoplastic tubules. Restaining with a different antibody or dilution was not attempted.

The patient did not receive chemotherapy. Currently, 7 years after the last surgery, the patient is alive and well, with no evidence of recurrence and eGFR is 73 ml/min/1.73 m^2^.

## Discussion and conclusions

According to the 2016 WHO classification, SDH-deficient RCC is composed of uniform cells characteristically displaying eosinophilic granular material intermixed with fewer cells exhibiting clear intracytoplasmic inclusions and bland centered nuclei [1, 2]. SDH-deficient renal carcinomas commonly entrap benign tubules and have cystic changes, as observed in the tumors in this patient [1, 2, 6, 7]. They lack the distinct granularity of oncocytes for which they may be mistaken. Immunohistochemistry is useful for diagnosis revealing a loss of expression of SDHB. The novel term SDH-deficient RCC should allow the correct classification of tumors with a specific pathogenesis, usually occurring in the context of hereditary conditions, and characteristic histological features, that, for the lack of a common term, had been previously assigned very different names in routine clinical practice and in the literature, as reflected in the 2009 description of the association of renal oncocytoma with *SDHB* mutations [8]. Prior to coining the term, most cases may have been misdiagnosed as clear cell RCC, chromophobe RCC, oncocytomas or hybrid tumors (Table 1). In fact, these authors concluded that “*SDHB* mutations should also be considered in the context of genetic testing when renal tumors, regardless of histopathology, present in families with other tumors consistent hereditary paraganglioma syndrome”. While we are not aware of bona fide cases of SDH-mutated RCC that weakly retained expression of SDHB, the phenomenon has been described in paragangliomas [9].

**Table 1:** Terms used to describe kidney tumors in patients with *SDHB* mutations before the current WHO 2016 classification coinage of the term SDH–deficient renal cell carcinoma [8]

Clear cell RCC (This case)	
Eosinophilic chromophobe RCC	
Papillary RCC (type II)	
Carcinoma not classifiable	
Mixed oncocytoma/ chromophobe carcinoma	
Oncocytoma	
Hybrid oncocytic tumor (This case)	

**Fig. 3:**
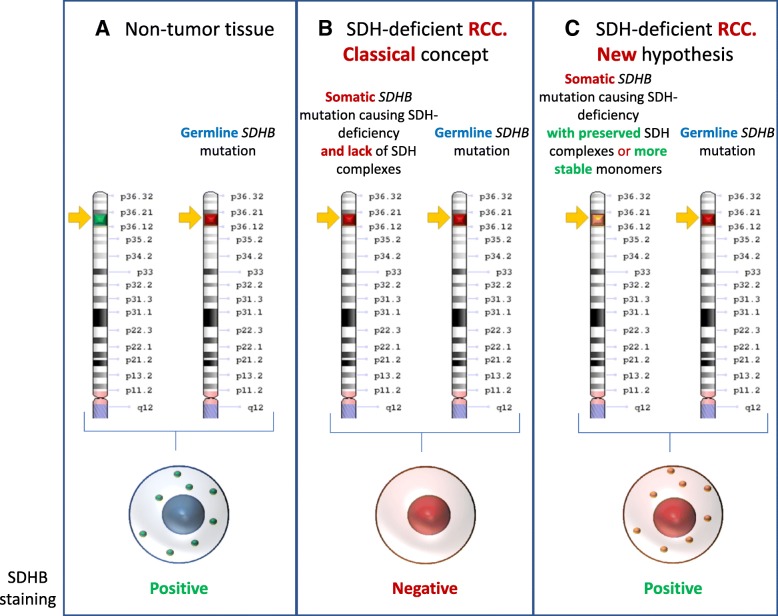
Hypothetical underlying molecular defect in SDHB immunostaining positive SDH–deficient RCC. Chromosome 1 is depicted. **a** Non-tumor tissue in patients with a germline pathogenic *SDHB* mutation. The disease is inherited in an autosomal dominant fashion and the wild-type allele is able to form functioning SDH complexes that are detected by immunostaining as granular (mitochondrial): cytoplasmic SDHB staining. **b** SDH–deficient RCC, classical concept. A somatic *SDHB* mutation associated with SDH–deficient RCC will result in non-formation of SDH complexes and in negative SDHB staining, as recognized by the 2016 WHO classification of tumors of the urinary system and male genital organs [1]. **c** SDH-deficient RCC, new hypothesis. Given the overall morphological similarity between the present case report and SDH–deficient RCC, we hypothesize that some somatic mutations may still result in SDH dysfunction but are associated with formation of SDH complexes and positive SDHB staining. The clinical impact would be that RCC that are stained positive for SDHB may still be observed in patients with *SDHB* mutations and that SDHB positivity in RCC does not exclude the existence of syndromic RCC due to *SDHB* mutations. Drawn by using the genome decoration page (https://www.ncbi.nlm.nih.gov/genome/tools/gdp)

SDH-deficient renal carcinoma is rare, less than 0.5% of all renal carcinomas [1, 2, 6, 7]. The added risk of RCC in patients with *SDHB* mutations was first described in 2008, when familial RCC was reported, even in the absence of a personal or family history of pheochromocytoma or head and neck paraganglioma, the most characteristics tumors associated to *SDHB* mutations, that cause pheochromocytoma/paraganglioma syndrome type 4 (PGL4) [10]. However, it was not until 2011 that the specific morphological features were reported and both the morphology and negative immunohistochemistry for SDHB in tumor cells proposed to allow the identification of kindreds with germline *SDHB* mutations [6]. Although the WHO classification of SDH-deficient RCC just refers to negative *SDHB* immunostaining [1, 2], a more detailed explanation of the criteria to consider SDHB immunostaining negative is provided by Gill et al. [1, 2, 6, 7]: A negative SDHB immunohistochemistry requires the entire tumor to demonstrate absent granular cytoplasmic (that is, mitochondrial) staining and the presence of readily identifiable internal positive controls in non-neoplastic cells. The present results are significant for the presence of clearly SDHB positive tumor cells displaying the characteristic staining pattern of SDHB positivity, in two tumors from the same patient with a bona fide *SDHB* genetic defect and concordant individual and familial phenotype, which otherwise displayed the morphological characteristics and clinical features of *SDHB* mutation-associated RCC. This illustrates the need to develop further markers of this type of RCC. Whether it also questions the need for homozygous *SDHB* deficiency for the pathogenesis of associated RCC may be discussed, since the molecular explanation for SDHB positivity in the tumor may theoretically be an acquired inactivating mutation in the wild-type *SDHB* allele that causes dysfunction but allows the complex to form (Fig. 3). As an alternative interpretation, a mutation may have resulted in expression of a dysfunctional SDHB subunit protein somehow rendered more stable as a monomer, rather than participating in assembly of an intact complex. Since we did not sequence the tumor, we cannot exclude this possibility.

The SDH complex is a key aerobic respiratory enzyme composed of four subunits: SDHA, SDHB, SDHC and SDHD, succinate dehydrogenase assembly factor 2 (SDHAF2) which form the mitochondrial complex 2, also known as succinate dehydrogenase/succinate–ubiquinone oxireductase or SDH. The complex is destabilized by the loss of any component, releasing the SDHB subunit into the cytoplasm where it degrades rapidly. The loss of SDHB observed by immunohistochemistry implies a double-hit inactivation, usually in the presence of a prior germline mutation, that prevents formation of the complex [1, 2, 6, 7]. It has been suggested that negative immunohistochemical staining for SDHB in tumors can be used as a surrogate marker for syndromic disease related to germline mutation of any of the components of the complex and it has been proposed that the term SDH-deficient can therefore be applied to all tumors which show loss of expression of SDHB, and SDH deficiency can be considered prima facie evidence of syndromic disease, usually dependent on a germline mutation [1, 2, 6, 7]. In this regard, rare cases of SDH-deficient RCC have also been associated with *SDHA* mutations (also with loss of SDHA expression by immunohistochemistry) and *SDHC* mutations have been reported.

The case provides lessons for other tumors. Thus, GIST, paraganglioma and pheochoromocytoma and the literature suggesting SDHD mutation in paraganglioma may be prone to difficult to interpret staining (diffuse blush negative may appear positive, like case 2 in this report). The reader should also be aware that tumors with very SDH-deficient RCC-like morphology, show retained expression of SDHB, but are in fact paradoxically FH-deficient [11, 12]. The implication of “missing” such a FH-mutated individual with HLRCC, which might present an identical morphology and SDH-retained phenotype as that described would be potentially even more serious than that of missing a SDH-deficient diagnosis.

False positive blush cytoplasmic staining may be challenging to differentiate from true mitochondrial positivity especially when non-neoplastic adjacent tubules are not available to be used as an internal control. Different results may be observed using different immunostaining platforms at the same dilution.

In individuals with SDHB deficiency, asynchronic SDHB-positive RCCs may occur, questioning the sensitivity of SDHB negative staining to detect individuals with germline SDH deficiency-associated multiple RCC. Since increasingly somatic tumor mutation profiling is performed, which might even obviate the need for an immunostaining, the WHO may reconsider its definition of SDHB-deficient tumor by adding a direct mutation profiling criterion.

## Additional file

Additional file 1: Genetic studies. (PDF 5 kb)

## Abbreviations

FCLN: folliculin gene
PGL4 syndrome: pheochromocytoma/paraganglioma syndrome type 4
RCC: Renal Cell Carcinoma
SDH: Succinate dehydrogenase
SDHA: Succinate dehydrogenase A
SDHAF2: succinate dehydrogenase assembly factor 2
SDHB: Succinate dehydrogenase B
SDHC: Succinate dehydrogenase C
SDHD: Succinate dehydrogenase D
WHO: World Health Organization
