# Supplementary material for: Positive/retained SDHB immunostaining in renal cell carcinomas associated to germline SDHB-deficiency: case report
Source: Diagn Pathol. 2019 May 15;14:42. doi: 10.1186/s13000-019-0812-6 (PMC6521540; doi:10.1186/s13000-019-0812-6)
Supplement: Supplementary file 1 — Genetic studies. (PDF 5 kb) [file 13000_2019_812_MOESM1_ESM.pdf]

Supplementary data

Genetic studies

The molecular study was performed using a panel of genes related to cancer. The enrichment of coding regions and the adjacent intronic regions was performed using a commercial kit (TrueSight Cancer), followed by high throughput sequencing (NextSeq500, Illumina), bioinformatics analysis (BaseSpace, Illumina) and annotation and interpretation of variants (VariantStudio v2,22, Illumina). The analysis initially focused on the FCLN gene, and no pathogenic variants were found. A further analysis of 16 genes related to renal cancer was performed and the heterozygous variant in the SDHB gene was found.
